# Frequency of Subclinical Hypothyroidism in Women With Polycystic Ovary Syndrome

**DOI:** 10.7759/cureus.17722

**Published:** 2021-09-04

**Authors:** Deepak Raj, FNU Pooja, Payal Chhabria, FNU Kalpana, Sameer Lohana, Kirshan Lal, Wajeeha Shahid, Sidra Naz, Simra Shahid, Dua Khalid

**Affiliations:** 1 Internal Medicine, Liaquat University of Medical and Health Sciences, Jamshoro, PAK; 2 Internal Medicine, Chandka Medical College, Larkana, PAK; 3 Internal Medicine, Jinnah Sindh Medical University, Karachi, PAK; 4 Internal Medicine, Shaheed Mohtarma Benazir Bhutto Medical University, Larkana, PAK; 5 Internal Medicine, Ghulam Muhammad Mahar Medical College, Sukkur, PAK; 6 Internal Medicine, University of Health Sciences, Lahore, PAK

**Keywords:** polycystic ovary syndrome, pcos, subclinical hypothyroidism, sch, hypothyroidism

## Abstract

Introduction: Polycystic ovary syndrome (PCOS) is a heterogeneous disorder characterized by hyperandrogenism and chronic anovulation. It may also influence thyroid hormones. Increasing evidence suggests that PCOS is linked with an increased prevalence of thyroid diseases such as nodular goiter, autoimmune thyroiditis, and subclinical hypothyroidism (SCH). Due to very limited global and regional data related to the prevalence of SCH in women with PCOS, we will determine the association between the two.

Methods: This case-control study was conducted in the endocrinology ward of a tertiary care hospital in Pakistan from March 2020 to April 2021. We enrolled 200 females between the ages of 18 and 30 years, with documented evidence of PCOS in the study. Further 200 females without PCOS were enrolled as the case group. After demographics were noted, blood was drawn from their cubital vein via phlebotomy and sent to the laboratory to assess for thyroid-stimulating hormone, free thyroxine, and free triiodothyronine.

Results: SCH was found to be more prevalent in participant with PCOS compared to participants without PCOS (43.5% vs. 20.5%; p-value: <0.00001). Increased weight (65.12 ± 5.62 kg vs. 60.02 ± 4.41 kg; p-value: <0.0001) and BMI (25.12 ± 2.51 kg/m^2^ vs. 22.51 ± 2.01 kg/m^2^; p-value: <0.0001) was significantly more in participants with PCOS compared to participants without PCOS.

Conclusion: Based on our findings, this study demonstrated the strong association of SCH in women with PCOS as compared to their normal counterparts. Therefore, the clinical implication is to maintain a high index of suspicion for signs and symptoms of SCH, and awareness is needed for such women to enhance the reproductive and clinical pregnancy outcomes.

## Introduction

Polycystic ovary syndrome (PCOS) is a heterogeneous disorder characterized by hyperandrogenism and chronic anovulation [1]. Primarily characterized by signs and symptoms of androgen excess and ovulatory dysfunction, it disrupts hypothalamic-pituitary-ovarian (HPO) axis function. Depending on diagnostic criteria, this disorder affects approximately 6%-20% of reproductive-aged women [2,3]. There are various hormonal changes associated with PCOS, including the tonic elevation of luteinizing hormone (LH) secretion as a regular feature of PCOS. Abnormal secretion of estrogen and high serum levels of free testosterone are also present [4].

PCOS may also influence thyroid hormones. There is increasing evidence to suggest that PCOS links to the increased prevalence of thyroid diseases such as nodular goiter, autoimmune thyroiditis, and subclinical hypothyroidism (SCH) [5,6]. A systematic review and meta-analysis demonstrated its strong association with an increased risk of SCH [6].

To date, there is very limited global and regional data related to the prevalence of SCH in women with PCOS. In this study, we will determine the association between the two.

## Materials and methods

This case-control study was conducted in the endocrinology ward of a tertiary care hospital in Pakistan from March 2020 to April 2021. We enrolled 200 females between the ages of 18 and 30 years, with documented evidence of PCOS in the study. Further 200 females without PCOS were enrolled as the case group. They were enrolled via consecutive convenient non-probability sampling techniques. Before proceeding with the data collection, ethical review board approval was taken from the institute and informed consent was taken from the participants. Participants with a history of cardiac disease, kidney disease, and on medication such as glucocorticoids and dopamine were excluded from the study.

After enrollment, participants’ demographics were noted in a self-structured questionnaire. Participants’ height and weight were measured to calculate body mass index (BMI). After demographics were noted, blood was drawn from their cubital vein via phlebotomy and sent to the laboratory to assess for thyroid-stimulating hormone (TSH), free thyroxine (FT4), and free triiodothyronine (FT3). SCH was defined as TSH levels between 5 and 10 mIU/L and normal FT3 and FT4 levels [7].

Data were analyzed using the Statistical Package for Social Sciences® software, version 23.0 (SPSS; IBM Corp., Armonk, NY, USA). Continuous variables such as age were presented as mean and standard deviation. Categorical variables were presented by percentages and frequencies. Mean hormone levels (TSH, FT3, and FT4) were compared using an independent t-test. The frequency of SCH was compared using the chi-square test. A p-value of less than 0.05 meant that there is a difference between the two groups and the null hypothesis is not valid.

## Results

Mean age and height were comparable between both groups. Increased weight (65.12 ± 5.62 kg vs. 60.02 ± 4.41 kg; p-value: <0.0001) and BMI (25.12 ± 2.51 kg/m2 vs 22.51 ± 2.01 kg/m^2^; p-value: <0.0001) was significantly more in participants with PCOS compared to participants without PCOS (Table 1).

**Table 1:** Demographics of participants with and without PCOS BMI: body mass index, cm: centimeter, kg: kilogram, kg/m^2^: kilogram per square meter, PCOS: polycystic ovary syndrome

Characteristics	Participants with PCOS (n=200)	Participants without PCOS (n=200)	P-value
Age (in years)	23.23 ± 3.13	23.54 ± 3.22	0.22
Height (in cm)	159.12 ± 10.21	158.21 ± 10.81	0.38
Weight (in kg)	65.12 ± 5.62	60.02 ± 4.41	<0.0001
BMI (kg/m^2^)	25.12 ± 2.51	22.51 ± 2.01	<0.0001

The level of TSH was significantly higher in participants with PCOS compared to participants without PCOS (5.01 ± 1.02 mIU/L vs. 3.42 ± 0.76 mIU/L; p-value: <0.00001). SCH was found to be more prevalent in participants with PCOS compared to participants without PCOS (43.5% vs. 20.5%; p-value: <0.00001) (Table 2).

**Table 2:** Comparison of thyroid profile of participants with and without PCOS FT3: free triiodothyronine, FT4: free thyroxine, mIU/L: milli-international units per liter, ng/dL: nanogram per deciliter, PCOS: polycystic ovary syndrome, pg/dL: picogram per deciliter, SCH: subclinical hypothyroidism, TSH: thyroid-stimulating hormone

Thyroid profile	Participants with PCOS (n=200)	Participants without PCOS (n=200)	P-value
TSH (mIU/L)	5.01 ± 1.02	3.42 ± 0.76	<0.0001
FT3 (pg/dL)	301.21 ± 87.65	312.18 ± 85.23	0.20
FT4 (ng/dL)	1.42 ± 0.34	1.47 ± 0.39	0.17
SCH (%)	87 (43.5%)	41 (20.5%)	<0.00001

## Discussion

Compared to participants without PCOS, our study indicates that women with PCOS had a significantly higher prevalence of SCH. TSH levels were observed to be significantly higher in participants with PCOS. Our study showed a higher prevalence of SCH in women with PCOS compared to other regional studies. In an Iranian study conducted by Enzevaei et al., SCH was demonstrated in approximately 25.5% of women with PCOS [8]. In line with this, another study conducted by Sinha et al. suggested that 22.5% of the Indian population with PCOS were declared to have SCH [9].

Several mechanisms have been suggested regarding the coexistence of PCOS with SCH. First, obesity and insulin resistance plays an important role in the mechanism of PCOS and SCH. Excessive BMI is known to enhance this effect. Abnormal fasting blood glucose (FBG) levels and insulin resistance were more likely in women who had SCH as compared to the ones without SCH [10,11]. Second, a compromised immune system seems to promote this interplay, as SCH can also result from autoimmune thyroiditis. Normally, the stimulatory activity of estrogen is neutralized by anti-inflammatory actions of progesterone levels but due to the anovulatory cycles in PCOS, the level of progesterone is nearly zero. This enhances the stimulatory activity of estrogen on the immune system, leading to a higher incidence of autoimmune diseases [12-14]. Third, several experiments have been conducted both in humans and animals to investigate the association between thyroid and ovary through immunohistochemistry, indicating that the luteal cells of mature corpora lutea may be involved in the synthesis of thyroid hormones [15]. The complications and consequences of SCH include increased risk of developing a variety of diseases like hyperlipidemia, impaired glucose metabolism, and cardiovascular diseases due to higher total cholesterol (TC), triglyceride (TG), and FBG in PCOS with SCH [16,17]. The strong association between SCH, PCOS, and psychological co-morbidities may also contribute to the psychological symptoms in such patients, such as anxiety and depression [18,19]. In addition to this, SCH during pregnancy results in several adverse maternal and neonatal outcomes, including premature rupture of membranes and neonatal death [20].

Due to the complications, greater awareness is needed for PCOS women with SCH. Metformin plays an important role in PCOS women with SCH by reducing serum TSH levels. Moreover, it also enhances the ovulation rate and reproductive outcomes in women with PCOS [21]. In addition, levothyroxine replacement therapy also reduces the miscarriage rate by improving the clinical pregnancy outcome in women with SCH [22]. 

Our studies add to limited data available on the prevalence of SCH in women with PCOS. However, there are few limitations as well. First, the study was conducted in a single institute hence the sample size was limited. Secondly, since it was a cross-sectional study relationship between the two variables could not be established.

## Conclusions

Our study indicates that SCH is significantly more prevalent in women with PCOS compared to women in the general population. PCOS in association with SCH can be responsible for clinical, biochemical, and metabolic changes, which result in adverse reproductive and pregnancy outcomes. Therefore, the clinical implication is to maintain a high index of suspicion for signs and symptoms of SCH, and awareness is needed for such women to enhance the reproductive and clinical pregnancy outcomes.
